# Therapeutic efficacy of artesunate in the treatment of uncomplicated *Plasmodium falciparum* malaria and anti-malarial, drug-resistance marker polymorphisms in populations near the China-Myanmar border

**DOI:** 10.1186/1475-2875-11-278

**Published:** 2012-08-16

**Authors:** Fang Huang, Linhua Tang, Henglin Yang, Shuisen Zhou, Xiaodong Sun, Hui Liu

**Affiliations:** 1 National Institute of Parasitic Diseases, Chinese Centre for Disease Control and Prevention; WHO Collaborating Centre for Malaria, Schistosomiasis and Filariasis; Key Laboratory of Parasite and Vector Biology, Ministry of Health, Shanghai 200025, PR China; 2 Yunnan Institute of Parasitic Diseases, Puer 665000, PR China

**Keywords:** Molecular markers, Artesunate, *Plasmodium falciparum*

## Abstract

**Background:**

The aim of this study was to evaluate the clinical outcome after seven-day artesunate monotherapy for uncomplicated *Plasmodium falciparum* malaria in Yingjiang County along the China-Myanmar border and investigate genetic polymorphisms in the *P. falciparum* chloroquine-resistance transporter (*pfcrt*), multidrug resistance 1 (*pfmdr1*), dihydrofolate reductase (*pfdhfr*), dihydropteroate synthase (*pfdhps*) and ATPase (*pfatp6*) genes.

**Methods:**

Patients ≥ one year of age with fever (axillary temperature ≥37.5°C) or history of fever and *P. falciparum* mono-infection were included. Patients received anti-malarial treatment with artesunate (total dose of 16 mg/kg over seven days) by directly observed therapy. After a 28-day follow-up, treatment efficacy and effectiveness were assessed based on clinical and parasitological outcomes. Treatment failure was defined as recrudescence of the original parasite and distinguished with new infection confirmed by PCR. Analysis of gene mutation and amplification were performed by nested polymerase chain reaction.

**Results:**

Sixty-five patients were enrolled; 10 withdrew from the study, and six were lost to follow-up. All but two patients demonstrated adequate clinical and parasitological response; 12 had detectable parasitaemia on day 3. These two patients were confirmed to be new infection by PCR. The efficacy of artesunate was 95.9%. The *pfcrt* mutation in codon 76 was found in all isolates (100%), and mutations in codons 71 and 72 were found in 4.8% of parasite isolates. No mutation of *pfmdr1* (codons 86 or 1246) was found. Among all samples, 5.1% were wild type for *pfdhfr*, whereas the other samples had mutations in four codons (51, 59, 108 and 164), and mutations in *pfdhps* (codons 436, 437, 540 and 581) were found in all isolates. No samples had mutations in *pfatp6* codons 623 or 769, but two new mutations (N683K and R756K) were found in 4.6% and 9.2% of parasite isolates, respectively.

**Conclusion:**

*Plasmodium falciparum* infection was associated with slow parasite clearance and suspected artemisinin resistance at the China-Myanmar border area. The prevalence of *pfcrt* 76 T and markers for SP resistance are still high. It should be strengthened further on parasite clearance time or clearance half life to confirm the resistance status, and molecular epidemiology should provide complementary information to assess the appropriateness of current policies based on artemisinin derivatives.

## Background

Malaria is a severe infectious disease, and drug resistance to anti-malarial drugs is a major public health problem worldwide [1]. In the last five years, there has been increasing concern about the emergence of resistance to artemisinin anti-malarials in Southeast Asia [2]. Several publications, including WHO reports, have provided evidence for the presence of *Plasmodium falciparum* tolerance/resistance to artesunate in populations on the Cambodia-Thailand border [3,4].

Falciparum malaria is now found in only two provinces in China: Yunnan province, which borders Myanmar, Laos and Vietnam, and Hainan province. Malaria control measures have been actively implemented in this area for more than 30 years with considerable success, and there are no local falciparum malaria cases in Hainan [5]. However, the malaria situation at the China-Myanmar border remains serious.

China was the first country to use artemisinins, but their wide-scale use in China began only in the early 1990s. The standard treatment regimen was 12 mg/kg artesunate or artemether over five days, which continued to be widely used until 2007. Since 2001, the WHO has recommended the use of artemisinin-based combination therapy in all areas where *P. falciparum* is resistant to other anti-malarial medicines to optimize therapeutic effectiveness and delay the emergence of resistance. In 2006, the WHO advocated a complete ban on artemisinin monotherapy for uncomplicated malaria [6]. The national drug policy of China was updated in 2009, and since then, the first-line drugs used to treat falciparum malaria has been artemisinin-based combination therapy (ACT), which includes dihydroartemisinin-piperaquine, artesunate-amodiaquine, artimisinin-naphthoquine phosphate and artemisinin-piperaquine [7].

Until now, ACT has been the only effective treatment for falciparum malaria. At a time when ACT is being rolled out in endemic malaria areas all over the world and is beginning to have an effect on morbidity and mortality in tropical Africa, the development and spread of ACT resistance would have grave consequences for global malarial control. Because of these concerns, national malaria programmes conduct systematic periodic *in vivo* malaria therapeutic efficacy studies in sentinel sites to monitor *P. falciparum* and *Plasmodium vivax* sensitivity to first-line, anti-malarial treatments.

Several genetic polymorphisms that can provide reliable data about the prevalence of drug resistance have been described in *P. falciparum* and *P. vivax*. The most relevant polymorphisms are presented below. The 76 T allele in the chloroquine-resistance transporter gene (*pfcrt*) is predictive of chloroquine and amodiaquine treatment failure [8-10]. The 86Y allele of the multidrug resistance gene 1 (*pfmdr1*) has been linked to chloroquine and amodiaquine resistance, and increased chloroquine inhibitory concentrations in *P. falciparum* have been linked with *pfcrt* 76 T [11]. The triple dihydrofolate reductase (*pfdhfr*) haplotype N51I/C59R/S108N has been associated with sulphadoxine-pyrimethamine (SP) treatment failure, and the addition of the dihydropteroate synthase (*pfdhps*) SNPs G437A and K540E produces highly resistant *P. falciparum*[12-16]. The sarco/endoplasmic reticulum Ca^2+^-ATPase ortholog of *P. falciparum* (*pfatp6*) was suggested to be involved in the mechanism of action and resistance of the parasite to artemisinins [17].

The aim of this study was to evaluate clinical outcome after seven-day artesunate monotherapy for uncomplicated *P. falciparum* malaria in Yingjiang County along the China-Myanmar border and to investigate the prevalence of genetic polymorphisms in *pfcrt*, *pfmdr1*, *pfdhfr*, *pfdhps* and *pfatp6*.

## Methods

### Study sites and design

The study was a one-arm prospective evaluation of clinical and parasitological response to directly observe treatment for uncomplicated malaria. The study was conducted in Yingjiang County, which is near Lazan City in Myanmar.

### Recruitment of patients

Patients over six months of age with fever (axillary temperature **≥**37.5**°**C) or history of fever in the previous 48 hours were included. The included patients had mono-infection with *P. falciparum*, parasitaemia levels between 250 and 100,000 asexual parasites/μl, no history of anti-malarial use in the previous 14 days and no signs of severe malaria or danger signs.

### Treatment of patients and follow-up

After written informed consent was obtained, a detailed medical history, clinical examination and both thick and thin blood films were performed. Artesunate was administered at a total dose of 16 mg/kg bw over seven days (first day: 4 mg/kg bw; second to seventh days: 2 mg/kg bw/day). Direct observed therapy (DOTS) was given for seven days by the health worker. Tablets of artesunate were obtained from the WHO (manufactured by Guilin Pharmaceutical Co., Ltd., Guilin, China). In case of vomiting within 30 minutes, the dose was repeated; if vomiting occurred within 30 minutes after the repeated dose, the patients were excluded from the study and referred for treatment at the healthcare facility.

Patients were followed up on days 1, 2, 3, 4, 5, 6, 7, 14, 21 and 28 for clinical and laboratory tests, which included axillary temperature measurement and thick blood smear preparation. If a patient could not be located on the day of the visit, the study staff attempted to locate the patient at his or her home. Patients who were not located within one day of the expected visit were considered lost to follow-up and excluded from the study.

Sample size was calculated based on a predicted treatment failure rate of 15% because the treatment failure rate of artesunate in the area was unknown. At a confidence level of 95% and a precision of approximately 5%, a minimum of 50 patients were required for the drug to be tested. At least 60 patients should be included to allow up to 20% loss to follow-up and withdrawals during the 28-day follow-up period.

Thick blood smears obtained on every visit day were stained with 10% Giemsa for 10 minutes and examined at 1,000× magnification by a trained microscopist to confirm the species and parasite density. The parasite density was determined by counting the number of asexual forms and gametocytes in 200 leukocytes. Parasitaemia was estimated by assuming 6,000 leukocytes/μl. A second microscopist blinded to the first result re-examined the thick blood smear, and in case of discrepancies greater than 50%, the smear was read by a third microscopist. The geometric mean of the two closest results was used as the parasitaemia value for each reading. When the number of asexual parasites was less than 10 per 200 white blood cells in follow-up smears, counting was performed in at least 500 white blood cells (ie, to completion of the field in which the 500th white blood cell was counted). A blood slide was considered to be negative when examination of 1,000 white blood cells revealed no asexual parasites. The presence of gametocytes on an enrolment or follow-up slide was noted, but this information did not contribute to the basic evaluation.

Genotype analysis was conducted to differentiate recrudescence (same parasite strain) from newly acquired infection (different parasite strain). This analysis is based on the extensive genetic diversity among the malaria parasite genes *msp1msp2* and *glurp*[18]. The genotypic profiles of pre- and post-parasite strains are compared.

### Outcome measures

Treatment efficacy and effectiveness were evaluated among observed patients based on clinical and parasitologic outcomes and study endpoints in accordance with WHO guidelines for *in vivo* efficacy monitoring [18]. The outcomes were classified as early treatment failure (ETF), late clinical failure (LCF), late parasitological failure (LPF) and adequate clinical and parasitological response (ACPR).

### Ethical considerations

The study was reviewed and approved by the ethical committee of the Chinese Centre for Disease Control and Prevention (China CDC). This study was also registered at the website [19] under the number ACTRN12610001008011.

### Molecular marker analysis

DNA was extracted from dried blood samples on filter papers using a QIAGEN mini kit and stored at −20°C until future use. Nested PCR [8,14,19,20] was used to amplify fragments of *pfcrtpfmdr1pfdhfrpfdhps* and *pfatp6*. Sequencing reactions were carried out using an ABI PRISM BigDye Terminator v3.1 Cycle Sequencing kit (Applied Biosystems, CA, USA) as specified by the manufacturer. The sequences of the amplicons were aligned with data published in the NCBI database by BLAST analysis.

## Results

### Study site

**Figure 1:**
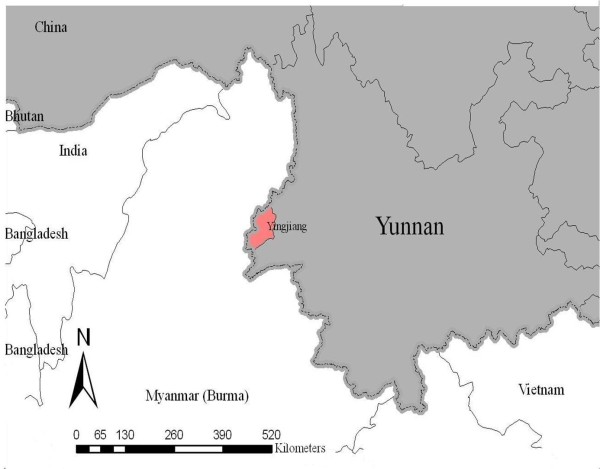
The location of Yingjiang County relative to neighbouring countries.

All of the patients were observed at the Rose Clinic in Lazan City, a small town on the Myanmar side of the border located 24°47′21.22″N and 97°33′21.05″ E (Figure 1). The Rose Clinic is close to the town of Nabang in Yingjiang County, China, and the two towns are separated by the Lazan stream. The town of Myitkina is located approximately 78 km north of Lazan City.

### Patient characteristics

**Figure 2:**
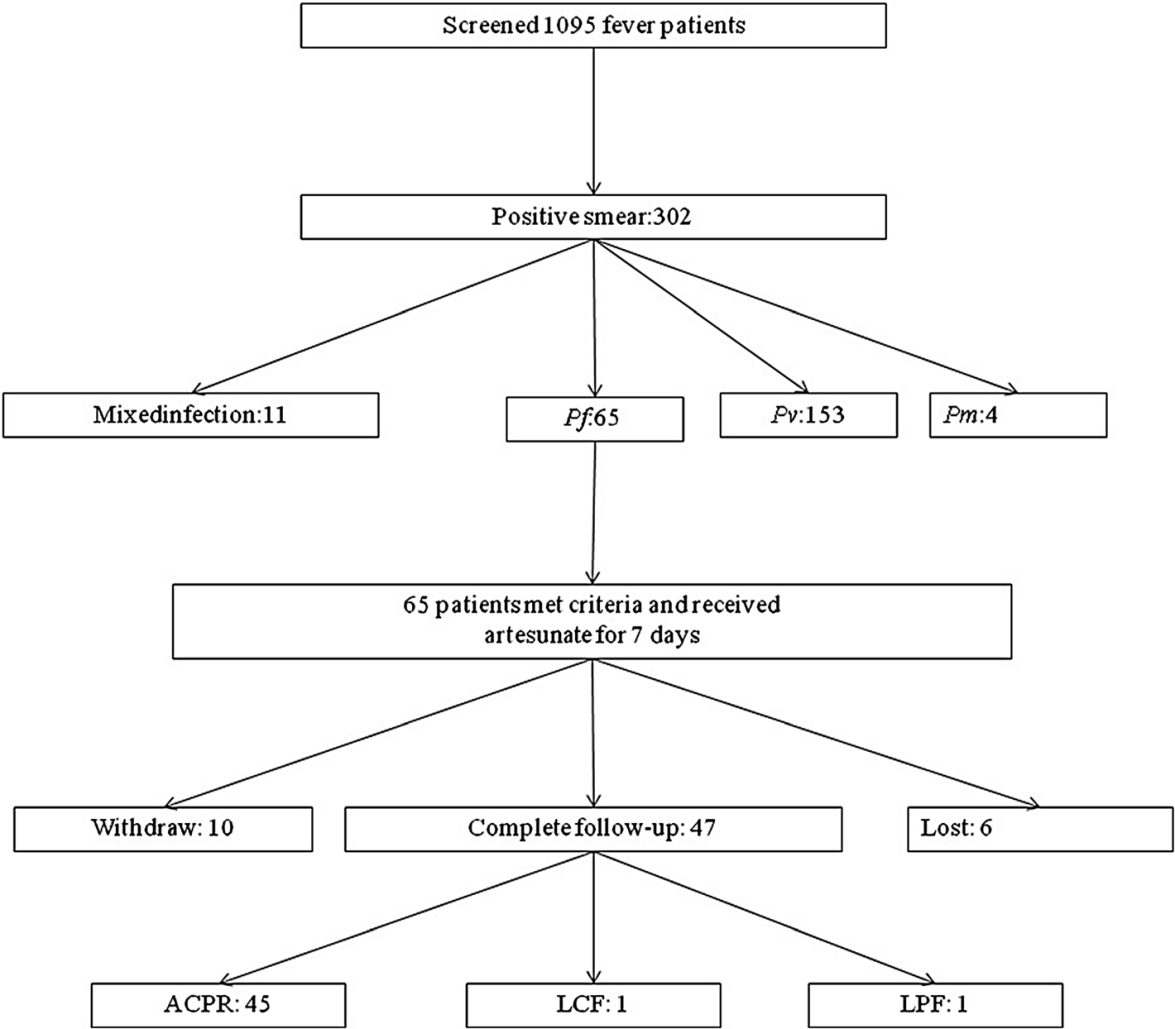
Patient recruitment and clinical outcomes.

A total of 1,095 febrile patients were screened for malaria by blood smear (microscopic examination). A total of 302 malaria patients were found, including 153 cases of *P. vivax*, 65 cases of *P. falciparum* malaria, four cases of *Plasmodium malariae* and 11 cases of mixed infection (Figure 2). All of the *P. falciparum* patients met the inclusion criteria.

Of the *P. falciparum* patients, 17 were female (26.2%) and 48 were male (73.8%). The age distribution was 3.0% under five years, 15.5% between five and 15 years and 81.5% >15 years. There was a mild predominance of young adult males. All of the patients had a fever in the 48 hours preceding presentation to the clinic, and 73.8% (48/65) exhibited an axillary temperature >37.5°C. The geometric mean parasite density was 69,535 parasites/μl. The probability of the patients with parasite on day 3 was correlated with the parasite densities on day 0, that is, the more parasite densities the more probability of positive on days 3 Table 1.

The patients’ ethnicity was also noted: 84% belonged to the Jingpo ethnic group, which is dominant on both sides of the border in this area; 13% were Bamar (Myanmar majority group); and the remaining 3% belonged to the local Lisu ethnic group. Among the 7% who were Chinese citizens, most were Han, and a few belonged to the Jingpo group.

### Clinical outcomes

Sixty-five patients were enrolled and received directly observed anti-malarial treatment with artesunate (total dose of 16 mg/kg over seven days); 10 withdrew from the study, and six were lost to follow-up. The efficacy of artesunate monotherapy was 95.9%.

All but two patients in the observed group demonstrated adequate clinical and parasitological response. The two patients with treatment failure were confirmed to have new infection by PCR. The allelic variants of *msp1*, *msp2* and *glurp* were genotyped and the results of the pre- and post-parasite strains of the two patients were shown in Table 2.

**Table 1:** **Characteristics of patients with *Plasmodium falciparum***

**Total**		**65**
Age (years)	Under 5	2
	5-15	10
	Adults (>15)	53
Gender	Male	48
	Female	17
History of fever (%)		100
Axillary temperature ≥ 37.5˚C		73.8 (48/65)
Geometric mean parasite density (/μl)		69,535

**Table 2:** The allele frequency of the pre- and post- treatment of the two “treatment failure” patients

**Sample**	**PCR Product Size(s) (bp)**					
	***msp2***		***Glurp***	***msp1***		
	**FC27**	**3D7/IC**		**RO33**	**K1**	**MAD20**
P1-D0		500	760	160		
P1-D14	320	500	960			220
P2-D0	400		600			160
P2-D14	350		820			220

Twelve patients had detectable parasitaemia on day 3. The geometric mean parasite density on Day 0 in the cases that were positive on day 3 was 89,519 asexual parasites per μl, indicating a strong association between day 3 positivity and high initial parasite density, as observed in other studies. The lowest day 0 parasitaemia associated with D3 positivity was 33,652 parasites/μl.

**Figure 3:**
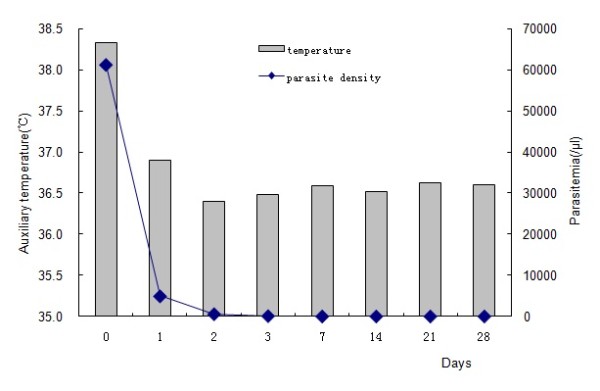
Average patient body temperature and parasite density on days 0, 1, 2, 3, 7, 14, 21 and 28 after artesunate treatment for seven days.

The average body temperature and parasite density of the patients decreased dramatically on days 0, 1, 2, 3, 7, 14, 21 and 28 after seven days of artesunate treatment (Figure 3).

### Molecular markers for drug resistance

#### *Pfcrt* and *pfmdr1*

A total of 63 samples were successfully amplified at positions 71, 72 and 76. Mutation in *pfcrt* codon 76 was found in all of the isolates (100%), and mutations in codons 71 and 72 were found in 4.8% of parasite isolates. No mutation in *pfmdr1* (codons 86 or 1246) was found.

#### *Pfdhfr* and *pfdhps*

**Figure 4:**
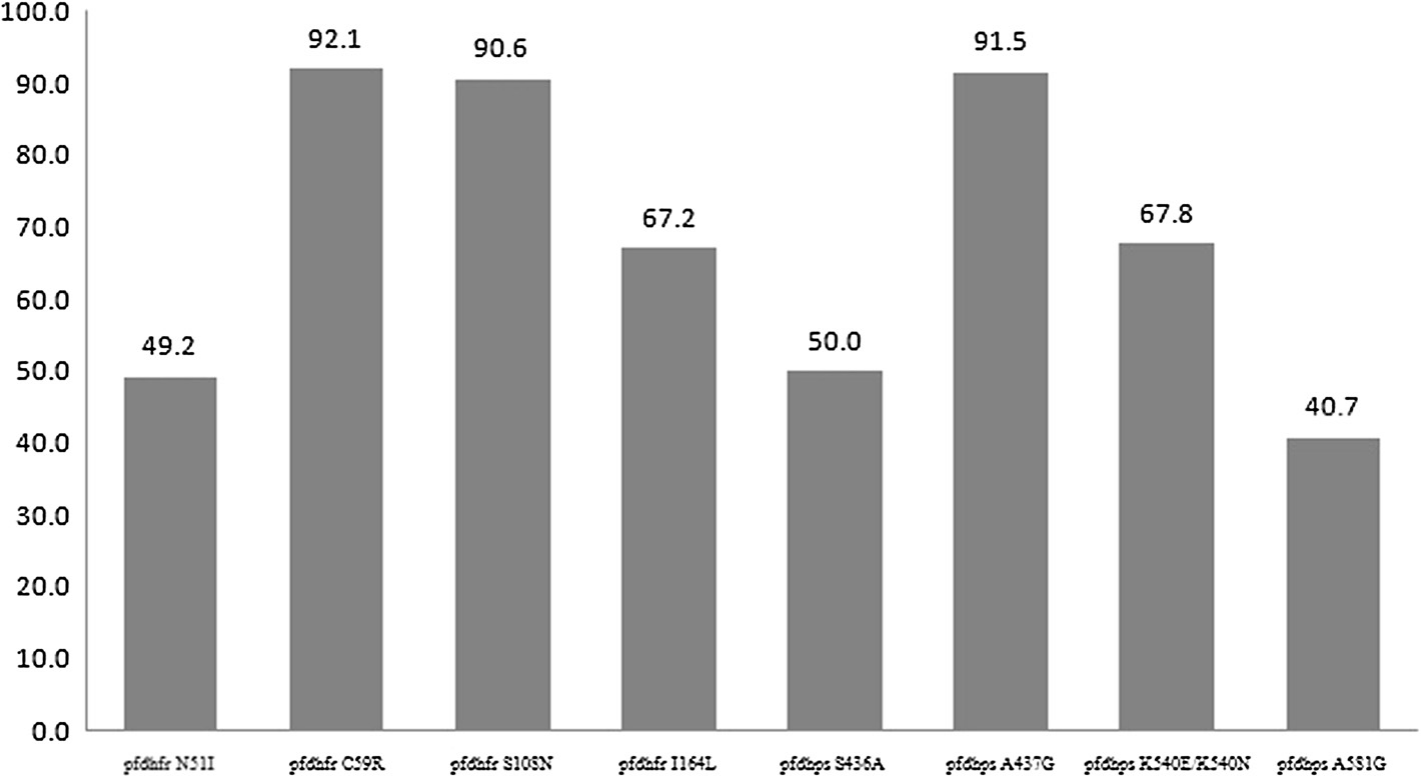
**Frequency of *pfdhfr *and *pfdhps *haplotypes in samples from Yingjiang County.**

The *pfdhfr* genes from 63 samples were successfully amplified and compared with the wild-type sequence. The frequencies of N51I, C59R, and I164L are shown in Figure 4. The most prevalent mutations were C59R and S108N, which were present in 92.1% and 90.6% of patients, respectively (Table 3). The mixed infections including quadruple (38.0%), triple (51.7) and double mutants (10.3%) were described in Table 3.

**Table 3:** **Prevalence of *pfdhfr *(codons 51, 59, 108 and 164) and *pfdhps *(codons 436, 437, 540 and 581) mutants**

**Gene**	**Mutation**	**Number**	**Frequency (%)**
***pfdhfr***	Quadruple mutants	22 (58)	38.0
	Triple mutants 51/59/108	9 (58)	15.5
	Triple mutants 59/108/164	21 (58)	36.2
	Double mutants 59/108	6 (58)	10.3
***pfdhps***	Quadruple mutants	13 (55)	23.6
	Triple mutants 436/437/581	8 (55)	14.5
	Double mutants 436/437	2 (55)	3.6
	Double mutants 436/540	1 (55)	1.8
	Double mutants 437/540	28 (55)	50.9
	Double mutants 437/581	3 (55)	5.5

For *pfdhps*, successful amplification was achieved in 59 samples, and mutations in codons 436, 437, 540 and 581 were found in all of the isolates (100%) including single, double, triple or quadruple mutants. The A437G and K540E mutants were most prevalent in *pfdhps*. Double mutants in *pfdhps* were found in 57.6% of samples and included 436/437, 436/540, 437/540, and 437/581 (Table 3). Except for one sample, all of the samples with the K540E mutation also had the A437G mutation.

#### *Pfatp6*

No mutation was found in *pfatp6* codons 623 or 769, but two new mutations (N683K and R756K) were found in 4.6% and 9.2% of parasite isolates, respectively.

## Discussion

Artemisinin-based combination therapy is recommended to slow the emergence and spread of drug resistance. This effect is achieved by the use of anti-malarial drugs with different mechanisms of action and the use of a fast-acting highly effective anti-malarial drug, such as DHA, in combination with a long-lasting anti-malarial drug, such as piperaquine. China was the first country to use artemisinin, and this drug was used for malaria treatment in China for almost 20 years (from the 1990s to 2007). In recent years, some reports confirmed artemisinin resistance in Southeast Asia, especially near Thailand-Cambodia border areas, but the status of artemisinin in China was unclear. The spread of artemisinin resistance from Cambodia to China or the *de novo* occurrence of artemisinin resistance in China is a serious concern. This study was designed to evaluate clinical outcome after seven-day artesunate monotherapy for uncomplicated *P. falciparum* malaria in Yingjiang County along the China-Myanmar border and investigate the prevalence of anti-malarial drug resistance markers. A parallel study evaluating DHA-PIP was conducted simultaneously in another county, and the results of that study will be published elsewhere.

Yunnan province is located in southern China, with a population of approximately 30 million people in 129 counties. The province borders Myanmar, the Lao People’s Democratic Republic and Vietnam. The border with Myanmar is 1,997 km long (as shown in Figure 1). Yingjiang County belongs to Dehong Prefecture with the total population of 288,691 people, which has the highest malaria incidence rate in China. The incidence in 2008 was 19.1 cases per 10,000, 38% of which were *P. falciparum*.

The majority of patients included in the study were from Myanmar or had been infected in a region of Myanmar close to the Chinese border. Among the 65 patients with *P. falciparum*, two had early treatment failure (4.1%), ie, confirmed re-infection. Therefore, this patient excluded as treatment failure with PCR correction until day 14. However, a key observation was the high rate of parasite positivity on day 3. A total of 18.5% of the enrolled patients were parasite positive on day 3. The initial parasite density was high, which might partially explain the high day 3 positivity rate, but the response still suggested a lowered susceptibility to artesunate, which was lower than the response rate observed in western Cambodia in recent years. It is impossible to say whether these findings reflect a spread from Cambodia or local factors. Local factors include long-term access to artemisinin (since at least the early or mid-1990s) and highly irregular treatment schedules in an area with continued moderate levels of transmission, as suggested by the predominance of malaria in young adults. Therefore, therapeutic efficacy tests should be continued, and the containment of artemisinin resistance in these areas should be prioritized.

*Pfcrt* and *pfmdr1* genes have been proposed as molecular markers of chloroquine resistance, and they also influence *P. falciparum* susceptibility to mefloquine, quinine, halofantrine and artemisinin [21-23]. According to the results of molecular marker tests, there was a high prevalence of mutation in codon 76 of *pfcrt*. As might be expected from the previous studies on *in vivo* and *in vitro* chloroquine responses in China [24], the isolates of *P. falciparum* examined in the present study frequently contained the *pfcrt* K76T mutation, especially in Yunnan province. Although China has not used chloroquine to treat *P. falciparum* for over 30 years, the stable and high prevalence of this mutation may be caused by the continued use of chloroquine as a first-line drug for *P. vivax* over several decades. Compared with the high prevalence of *pfcrt* mutations, there was no mutation in codons 86 or 1246 of *pfmdr1* in any sample. This result was consistent with other researchers’ findings [25-27]. Until now, the relationship between the Y86 mutation and chloroquine resistance has not been confirmed, and there have been many conflicting results. The results in this research may indicate that *P. falciparum* in China is still sensitive to mefloquine, quinine, halofantrine and artemisinin.

The effects of *pfdhfr* and *pfdhps* mutations on the mechanism of resistance to SP drugs have been well described. Pyrimethamine was used for the radical treatment of *P. vivax* in combination with primaquine 40 years ago [28]. Additionally, pyrimethamine was added to salt for prophylaxis in the 1980s [29]. Pyrimethamine plus primaquine has always been recommended as prophylaxis for specific populations in China [7] and sulphadoxine-pyrimethamine were used in Myanmar in 1980’s and the resistance at various levels were common throughout the country in the beginning of 1990’s [30]. This study found that the prevalence of quadruple, triple and double mutants at the China-Myanmar border was high, which suggested that these parasites are still resistant to anti-folate anti-malarial drugs in China, two decades after SP was abandoned as a first-line anti-malarial [31]. This research also confirmed that the *pfdhps* A437G mutation confers resistance to sulphadoxine and is often coupled with the K540E mutant allele.

The mechanism of action of artemisinin remains controversial. One of its proposed mechanisms involves an interaction with the sarcoplasmic reticulum Ca^2+^ ATPase6. The analysis of naturally occurring *pfatp6* polymorphisms in field isolates suggested that a polymorphism at codon 769 may be associated with the reduced susceptibility of these isolates to artemether *in vitro*[17]. In line with other investigators, there was any polymorphism in codons 263 or 769, which are described as the key amino acids in the interaction between *pfatp6* and artemisinins [32]. However, two new mutations (N683K and R756K) were found in parasite isolates in this study, and the mutation of position 683 has not been reported previously [33]. The mutations observed in this study and in previous studies [34-36] could be indirectly implicated in this interaction if they are associated with artemisinin susceptibility. But one recent study [37] on emergence of artemisinin-resistant malaria on the western border of Thailand showed the proportion of variation in parasite clearance attributable to parasite genetics increased doubling compared with year 2001–2004 and year 2007–2010. Considering the development of artemisinin combination therapy and the possible implication of *pfatp6* in artemisinin resistance, all of the polymorphisms in this gene should be carefully monitored.

## Conclusion

Slow parasite clearance and suspected artemisinin resistance was observed in Yingjiang County at the China-Myanmar border area, therefore it should be strengthen further on parasite clearance time or clearance half life to confirm the resistance status. And now Yunnan province of China, as part of Greater Mekong Sub-region, has already involved in “**Greater Mekong Sub-region Programme for Artemisinin Resistance Containment**”, which will be launching this year or next year.

Also, this study provided basic data on artemisinin resistance in this area, which will be helpful for other countries in the Mekong sub-region. Moreover, molecular epidemiology should be continued as routine surveillance and to provide complementary information to assess the appropriateness of current policies based on artemisinin derivatives.

## Competing interests

The authors hereby certify that no conflict of interest of any kind occurred in the framework of this study.

## Authors’ contributions

FH was responsible for the molecular genetic studies, participated in the field work, and drafted the manuscript. LT was responsible for the overall study design and was involved in all stages of the study, including field work. HY was involved in the design of the field work. SZ contributed to the molecular genetic studies and data analysis. XS and HL contributed to the field study, patient follow-up and data analysis. All of the authors read and approved the final manuscript.
